# Influence of marital status on overall survival in adult patients with chordoma: a SEER-based study

**DOI:** 10.1186/s13018-020-01803-6

**Published:** 2020-07-23

**Authors:** Chao Tang, Ruiliang Wang, Qingguo Lu, Shantao Wang, Gen Jia, Pengfei Cao, Xinfa Nie, Hailong Zhang

**Affiliations:** 1 Pain Department, Pizhou City People’s Hospital, Xuzhou Medical University, Xuzhou City, 200032 Jiangsu Province China; 2 Nursing Department, Xijing Hospital, Air Force Medical University, Xi’an City, 710032 Shanxi Province China; 3 Trauma Center, Pizhou City People’s Hospital, Xuzhou Medical University, Xuzhou City, 200032 Jiangsu Province China; 4 Orthopedic Department, People’s Hospital of Putuo District, Tongji University School of Medicine, Shanghai, 200060 China

**Keywords:** Chordoma, Marital status, Overall survival, Prognosis, SEER

## Abstract

**Background:**

As a rare primary bone tumor, no studies have reported the relationship between prognosis and marital status in patients with chordoma.

**Methods:**

We classified patients with chordoma identified from the Surveillance, Epidemiology, and End Results (SEER) database from 1975 to 2016 into four groups: married, divorced/separated, widowed, and single groups. Kaplan-Meier curves with log-rank test and Cox regression were used to analyze the effect of marital status on overall survival (OS).

**Results:**

A total of 1080 patients were included in the study: 700 (64.8%) were married, 88 (8.1%) were divorced/separated, 78 (7.2%) were widowed, and 214 (19.8%) were single. Among the 4 groups, the 5-year OS (45.2%), 10-year OS (12.5%), and median OS (56.0 months) were the lowest in the widowed group. After including age, sex, primary site, marital status, disease stage, tumor size, histological type, and treatment pattern, multivariate analysis showed that marital status was still an independent risk factor for patients with chordoma, and widowed patients had the lowest OS (hazard ratio [HR] 1.71; 95% confidence interval [CI] 1.25–2.33, *p* < 0.001) compared with married patients. Similar results were observed after stratifying the primary site and disease stage.

**Conclusion:**

Marital status was an independent prognostic indicator for adult patients with chordoma, and marital status was conducive to patient survival. Compared with married patients, widowed patients have a higher risk of death.

## Introduction

Chordomas are rare bone tumors that accounts for approximately 20% of primary spinal tumors and 3% of all bone tumors [1]. It is a rare and locally destructive tumor that originates from the residual tissue of the embryonic spinal cord structure and can occur anywhere along the midline bone, especially the slope of the skull base, the saddle area, and the tail of the spine [2, 3]. A survey of European and American populations showed that the incidence rate of chordomas was approximately 0.08/100,000, which was slightly higher in males [4]. Although chordomas grow slowly, due to their aggressive and easy metastasis, chordoma can infiltrate the surrounding bone structure [5]. Due to its high recurrence rate, which seriously affects the survival rate and the quality of life of patients, the total 5-year survival rate was only approximately 67% [6].

Many factors affect the prognosis of patients with chordoma. Previous studies have shown that surgical margin and distant metastasis were independent prognostic factors in patients with chordoma [7, 8]. In addition, patient age, histological type, and tumor size may also affect the survival of patients with chordoma [7, 9, 10].

Marital status has always been closely related to the prognosis of cancer. Many studies have confirmed that marital status may affect the prognosis of various tumors, including osteosarcoma [11], chondrosarcoma [12], penile cancer [13], and breast cancer [14]. However, retrospective or prospective studies have not been conducted to report whether marital status affects the survival of adult patients with chordoma. Therefore, the purpose of this study was to investigate the effect of marital status on the survival of patients with chordoma according to the Surveillance, Epidemiology, and End Results (SEER) database.

## Materials and methods

### Patient selection

The patients we studied were selected from the Surveillance Epidemiology and End Results (SEER) database funded by the National Cancer Institute. The SEER database covers approximately 28% of the USA population and includes demographic information and cancer characteristics, such as year of diagnosis, age, origin, race, insurance, marital status, primary tumor location, income status, tumor grade, disease stage, histological type, tumor-node-metastasis (TNM) stage, treatment modality, and survival time [15]. The National Cancer Institute’s SEER*Stat software (version 8.3.6; SEER 18 Regs Custom Data (with additional treatment fields), November 2018 Sub (1975–2016 varying) database) was used in this study. We included 1521 patients diagnosed with chordoma between 1 January, 1975 and 31 December 2016 based on the *International Classification of Diseases for Oncology* (9370: chordoma, NOS; 9371: chondroid chordoma; 9372: dedifferentiated chordoma).

The exclusion criteria were as follows: (a) not one primary tumor only (*n* = 298); (b) primary site code not 41.0, 41.2, or 41.4 (*n* = 9); (c) marital status unknown or domestic partner (*n* = 61); (d) unknown survival time (*n* = 2); and (e) patients under 18 years of age (*n* = 71). Finally, based on the above screening criteria, we were left with 1080 eligible patients diagnosed with chordoma.

### Study variables

Variable definition information about year of diagnosis, age at diagnosis, sex, primary site, marital status, disease stage, tumor size, histological type, treatment pattern (surgery [16], radiotherapy, chemotherapy), and survival time can be found in the SEER database. The starting point of the follow-up was the date of diagnosis of chordoma. The overall survival (OS) time is the length of time from the date of diagnosis to the end of the patient’s follow-up or death.

### Statistical analysis

Chi-square analysis was performed to evaluate the clinical characteristics of the four marital statuses in patients with chordoma. Kaplan-Meier curves were used to estimate the factors related to the OS, 5-year OS, and 10-year OS of patients with chordoma, and the log-rank test was used to analyze the difference between the curves. Univariate and multivariate Cox regression models were performed to estimate the hazard ratios (HRs) and 95% confidence intervals (CIs) to analyze independent prognostic factors associated with patients with chordoma. All statistical analyses used Statistical Package for the Social Sciences software (version 24.0; SPSS, Chicago, USA) and R version 3.5.3 (R Foundation for Statistical Computing, http://www.r-project.org/). The Survminer package included in Kaplan-Meier analysis with log-rank testing was applied to conduct the survival data analysis and visualization (Drawing Survival Curves using ‘ggplot2’ [R package survminer version 0.2.0]). Univariate Cox proportional hazards regression and multivariate Cox proportional hazards regression with the Wald test were performed to determine risk factors associated with overall mortality and cancer-specific mortality. Statistical significance was considered when the *p* value is ≤ 0.05 (both sides).

## Results

### Demographic and clinicopathologic characteristics of patients with chordoma

**Fig. 1:**
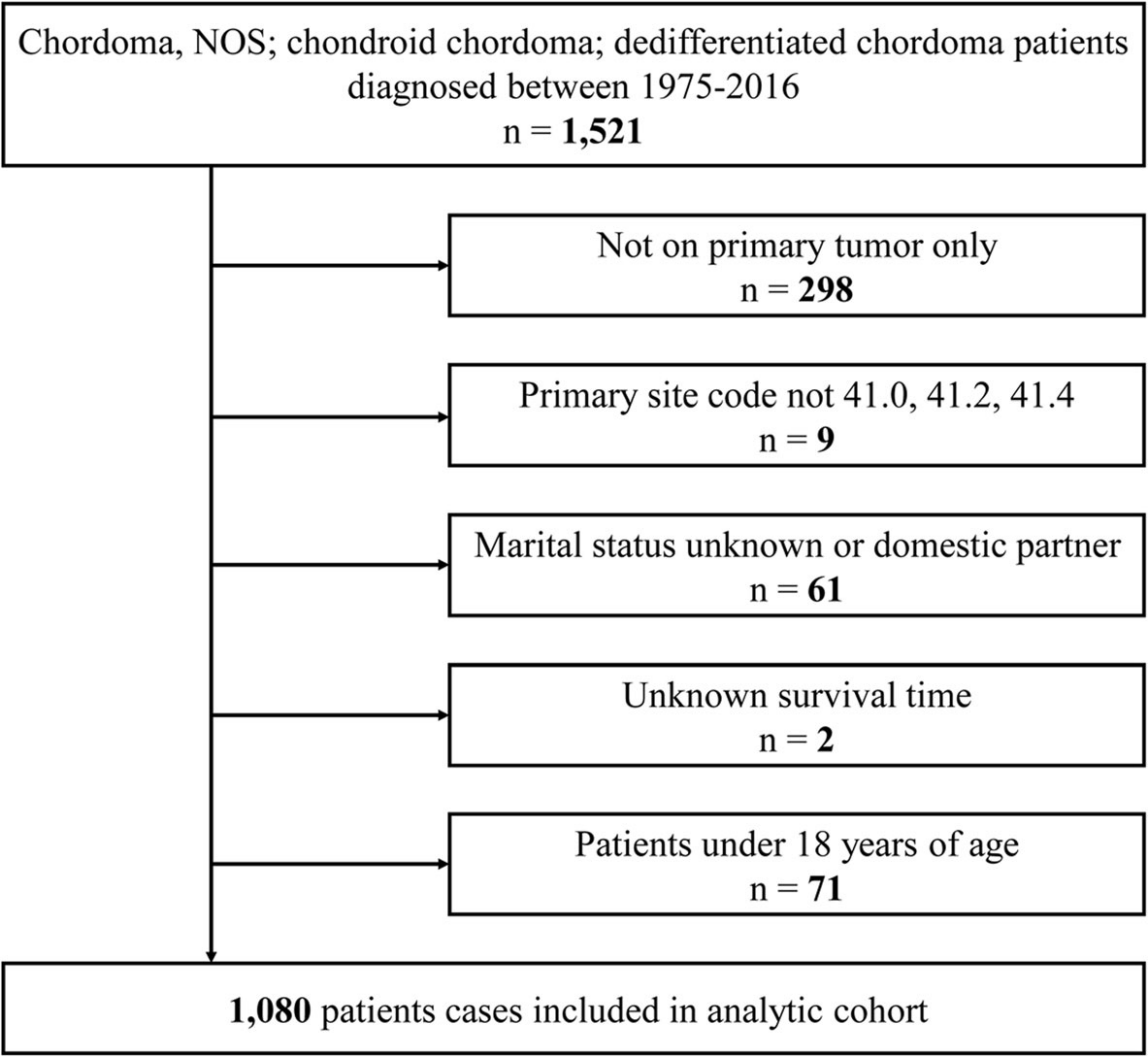
Schematic flow diagram of the inclusion and exclusion criteria for our study cohort

According to the inclusion and exclusion criteria in Fig. 1, our study included a total of 1080 eligible patients with chordoma from 1975 to 2017. The number of married, divorced/separated, widowed, and single group were 700 (64.8%), 88 (8.1%), 78 (7.2%), and 214 (19.8%), respectively. Table 1 shows the clinical characteristics and demographic of all adult patients with chordoma. The chi-square test showed that there were significant differences in the five variables of diagnosis year (*p* = 0.014), age at diagnosis (*p* < 0.001), sex (*p* < 0.001), primary site (*p* = 0.019), and surgery (*p* < 0.001). With the increase in years, the proportion of patients with chordoma also increased. In the whole cohort, the majority of patients were male (59.9%), the primary site was bones of the skull and face and associated joints (40.4%), and localized stage predominated (40.9%). In addition, the percentages of older (> 60 years, 94.9%), female (73.1%), and non-surgery (37.2%) patients in the widowed group were also the highest.

**Table 1:** Baseline demographic and clinical characteristics of chordoma patients in our study

Characteristic	Total no. (%)	Married	Divorced/separated	Widowed	Single	*p* value
		No. (%)	No. (%)	No. (%)	No. (%)	
Total	1080	700 (64.8)	88 (8.1)	78 (7.2)	214 (19.8)	
Year of diagnosis						**0.014**
1975–1988	98 (9.1)	70 (10.0)	4 (4.5)	12 (15.4)	12 (5.6)	
1988–2002	262 (24.3)	181 (25.9)	19 (21.6)	20 (25.6)	42 (19.6)	
2003–2016	720 (66.7)	449 (64.1)	65 (73.9)	46 (59.0)	160 (74.8)	
Age at diagnosis						**< 0.001**
< 40	242 (22.4)	131 (18.7)	18 (20.5)	0 (0.0)	93 (43.5)	
40–60	412 (38.1)	294 (42.0)	35 (39.8)	4 (5.1)	79 (36.9)	
> 60	426 (39.4)	275 (39.3)	35 (39.8)	74 (94.9)	42 (19.6)	
Sex						**< 0.001**
Male	647 (59.9)	441 (63.4)	46 (52.3)	21 (26.9)	136 (63.6)	
Female	433 (40.1)	256 (36.6)	42 (47.7)	57 (73.1)	78 (36.4)	
Primary site						**0.019**
Bones of skull and face and associated joints	436 (40.4)	285 (40.7)	34 (38.6)	17 (21.8)	100 (46.7)	
Vertebral column	279 (25.8)	179 (25.6)	25 (28.4)	27 (34.6)	48 (22.4)	
Pelvic bone, sacrum, coccyx, and associated joints	365 (33.8)	236 (33.7)	29 (33.0)	34 (43.6)	66 (30.8)	
Disease stage						0.587
Localized	442 (40.9)	288 (41.1)	38 (43.2)	30 (38.5)	86 (40.2)	
Regional	454 (42.0)	290 (41.4)	41 (46.6)	32 (41.0)	91 (42.5)	
Distant	90 (8.3)	55 (7.9)	5 (5.7)	7 (9.0)	23 (10.7)	
Unstaged	94 (8.7)	67 (9.6)	4 (4.5)	9 (11.5)	14 (6.5)	
Tumor size						0.084
< 5 cm	357 (33.1)	229 (32.7)	32 (36.4)	18 (23.1)	78 (36.4)	
5–10 cm	256 (23.7)	155 (22.1)	21 (23.9)	21 (26.9)	59 (27.6)	
> 10 cm	101 (9.4)	64 (9.1)	10 (11.4)	5 (6.4)	22 (10.3)	
Unknown	366 (33.9)	252 (36.0)	25 (28.4)	34 (43.6)	55 (25.7)	
Histological type						0.953
Conventional chordoma	1019 (94.4)	659 (94.1)	83 (94.3)	75 (96.2)	202 (94.4)	
Chondroid chordoma	54 (5.0)	37 (5.3)	4 (4.5)	3 (3.8)	10 (4.7)	
Dedifferentiated chordoma	7 (0.6)	4 (0.6)	1 (1.1)	0 (0.0)	2 (0.9)	
Surgery						**< 0.001**
Surgery not performed	177 (16.4)	99 (14.1)	20 (22.7)	29 (37.2)	29 (13.6)	
STR	452 (41.9)	295 (42.1)	38 (43.2)	23 (29.5)	96 (44.9)	
GTR	301 (27.9)	199 (28.4)	23 (26.1)	12 (15.4)	67 (31.3)	
Unknown extent of resection	150 (13.9)	107 (15.3)	7 (8.0)	14 (17.9)	22 (10.3)	
Radiotherapy						0.734
Yes	544 (50.4)	354 (50.6)	48 (54.5)	40 (51.3)	101 (47.7)	
No	536 (49.6)	346 (49.4)	40 (45.5)	38 (48.7)	112 (52.3)	
Chemotherapy						0.639
Yes	40 (3.7)	24 (3.4)	3 (3.4)	2 (2.6)	11 (5.1)	
No	1040 (96.3)	676 (96.6)	85 (96.6)	76 (97.4)	203 (94.9)	

Note: *p* value < 0.05 are shown in bold

*Abbreviations: STR* subtotal resection, *GTR* gross total/radical resection

Percentages may not total 100 because of rounding

### Survival of patients with chordoma

**Fig. 2:**
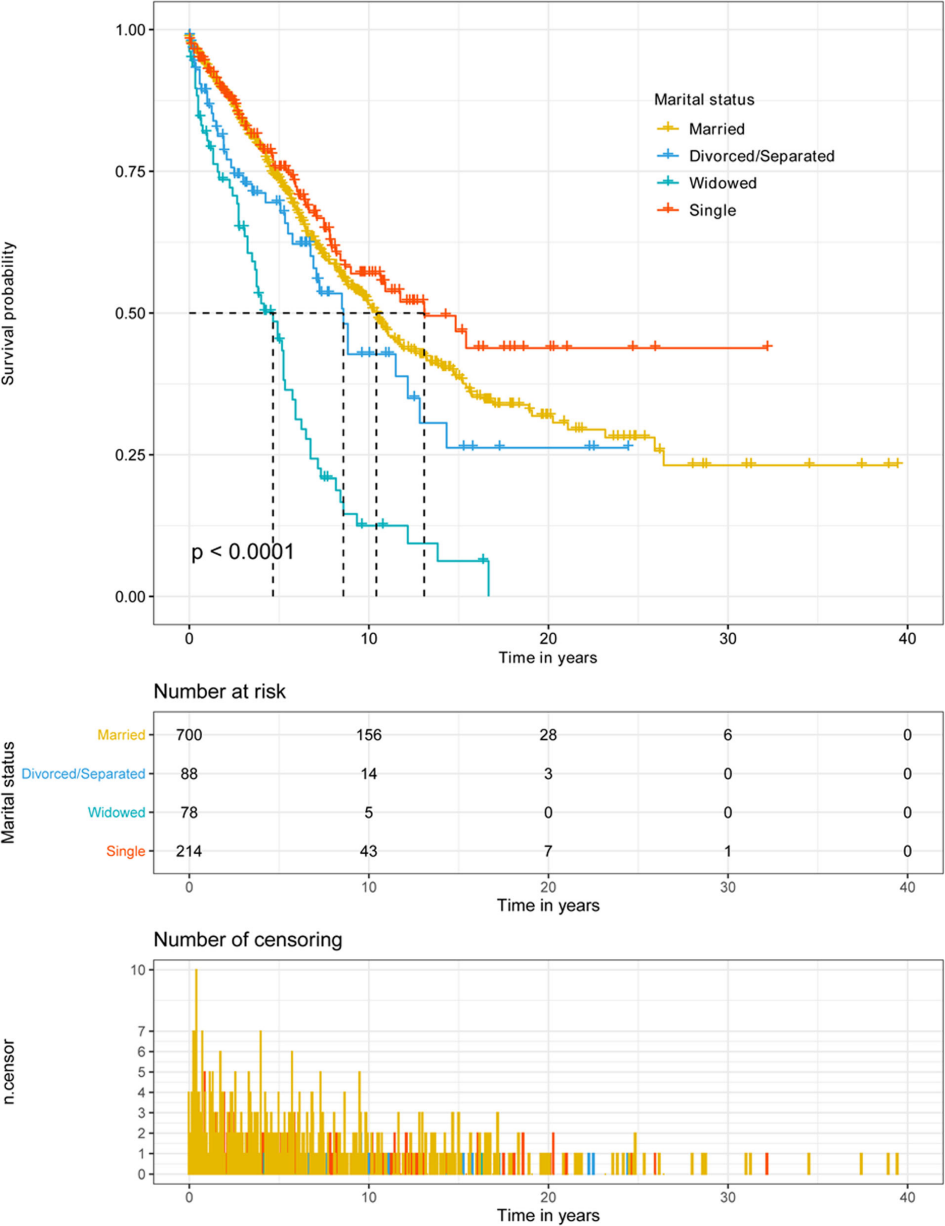
Kaplan-Meier survival curves according to marital status in patients with chordoma

**Fig. 3:**
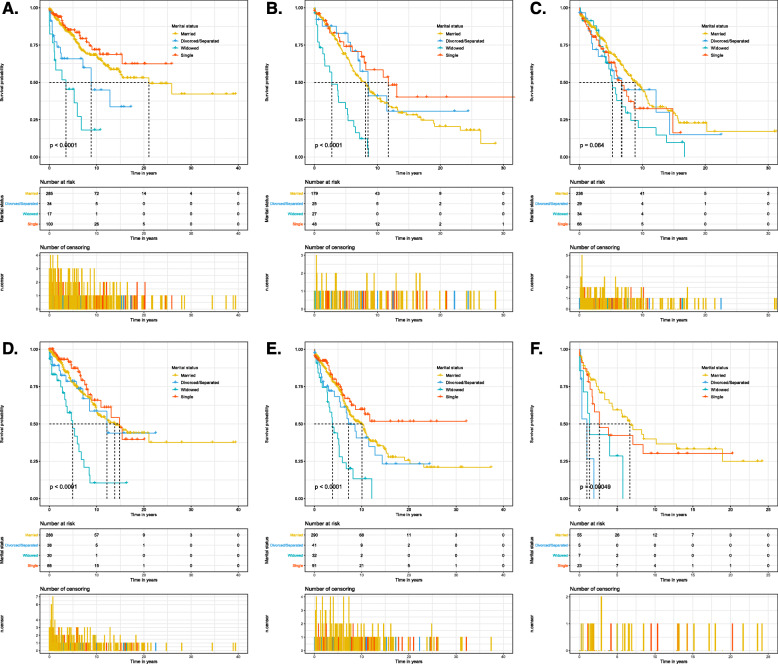
Overall survival curves of patients with chordoma according to marital status at different primary sites and disease stages. **a** Bones of the skull and face and associated joints. **b** Vertebral column. **c** Pelvic bone, sacrum, coccyx, and associated joints. **d** Localized stage. **e** Regional stage. **f** Distant stage

By analyzing the Kaplan-Meier curve with a log-rank test, we found that age at diagnosis (*p* < 0.001), marital status (*p* < 0.001), primary site (*p* < 0.001), disease stage (*p* < 0.001), tumor size (*p* < 0.001), histological type (*p* = 0.002), surgery (*p* < 0.001), and chemotherapy (*p* = 0.001) were associated with OS (Table 2). The 5-year OS and 10-year OS of married, divorced/separated, widowed, and single patients were 73.7% and 51.5%, 69.5% and 42.8%, 45.2% and 12.5%, and 75.6% and 57.0%, respectively, and the median survival times of married, divorced/separated, widowed, and single patients were 125.0 months, 103.0 months, 56.0 months, and 157.0 months, respectively (Fig. 2). Widowed patients had the lowest 5-year OS, 10-year OS, and median overall survival time, while single patients had the highest 5-year OS, 10-year OS, and median overall survival time. After stratifying the primary site and disease stage, we still observed similar results (Table 3 and Fig. 3).

**Table 2:** Kaplan–Meier analysis overall survival for chordoma patients

Characteristic	5-year overall survival, %	10-year overall survival, %	Median overall survival (months)	Kaplan-Meier	
				Log rank *χ2* test	*p* value
Age at diagnosis				164.433	**< 0.001**
< 40	83.8	74.4	–		
40–60	82.8	58.7	138.0		
> 60	54.3	25.2	68.0		
Sex				2.585	0.108
Male	70.5	45.9	105.0		
Female	72.9	52.0	132.0		
Marital status				66.240	**< 0.001**
Married	73.7	51.5	125.0		
Divorced/separated	69.5	42.8	103.0		
Widowed	45.2	12.5	56.0		
Single	75.6	57.0	157.0		
Primary site				41.055	**< 0.001**
Bones of skull and face and associated joints	79.2	65.1	253.0		
Vertebral column	66.7	37.9	90.0		
Pelvic bone, sacrum, coccyx, and associated joints	66.4	40.1	91.0		
Disease stage				29.554	**< 0.001**
Localized	77.6	55.3	147.0		
Regional	71.6	48.2	105.0		
Distant	49.7	32.7	53.0		
Unstaged	65.2	39.0	89.0		
Tumor size				45.181	**< 0.001**
< 5 cm	82.3	70.9	243.0		
5–10 cm	70.5	45.9	106.0		
> 10 cm	55.9	32.1	70.0		
Unknown	67.0	41.0	94.0		
Histological type				12.265	**0.002**
Conventional chordoma	71.5	47.6	110.0		
Chondroid chordoma	76.6	72.8	-		
Dedifferentiated chordoma	28.6	28.6	14.0		
Surgery				97.790	**< 0.001**
Surgery not performed	48.3	26.0	56.0		
STR	78.1	58.5	154.0		
GTR	82.9	59.3	178.0		
Unknown extent of resection	59.1	31.9	80.0		
Radiotherapy				0.140	0.708
Yes	72.7	47.1	106.0		
No	70.0	49.7	120.0		
Chemotherapy				11.445	**0.001**
Yes	47.9	28.1	51.0		
No	72.4	49.4	119.0		

Note: *p* value < 0.05 are shown in bold

*Abbreviations: STR* subtotal resection, *GTR* gross total/radical resection

**Table 3:** Kaplan–Meier analysis overall survival for chordoma patients based on primary site and disease stage

Characteristic	5-year overall survival, %	10-year overall survival, %	Median overall survival (months)	Kaplan-Meier	
				Log rank *χ2* test	*p* value
Primary site				35.462	**< 0.001**
Bones of skull and face and associated joints					
Married	81.0	68.3	253.0		
Divorced/separated	65.8	44.9	106.0		
Widowed	45.3	18.1	42.0		
Single	85.0	71.8	–		
Vertebral column				38.754	**< 0.001**
Married	68.2	38.9	97.0		
Divorced/separated	82.8	40.9	102.0		
Widowed	32.5	0.0	33.0		
Single	74.1	58.3	141.0		
Pelvic bone, sacrum, coccyx, and associated joints				7.278	0.064
Married	69.6	44.9	106.0		
Divorced/separated	62.6	45.0	81.0		
Widowed	54.7	19.6	63.0		
Single	63.1	32.4	80.0		
Disease stage					
Localized				33.341	**< 0.001**
Married	78.1	57.8	166.0		
Divorced/separated	78.5	58.5	146.0		
Widowed	47.0	10.4	59.0		
Single	87.2	65.8	178.0		
Regional				31.648	**< 0.001**
Married	74.4	50.3	121.0		
Divorced/separated	68.4	40.7	87.0		
Widowed	40.0	13.3	46.0		
Single	76.5	59.7	–		
Distant				17.771	**< 0.001**
Married	59.4	39.9	80.0		
Divorced/separated	0.0	0.0	12.0		
Widowed	28.6	0.0	16.0		
Single	42.3	30.2	32.0		

Note: *p* value < 0.05 are shown in bold

### Identification of prognostic factors of the OS of patients with chordoma

**Fig. 4:**
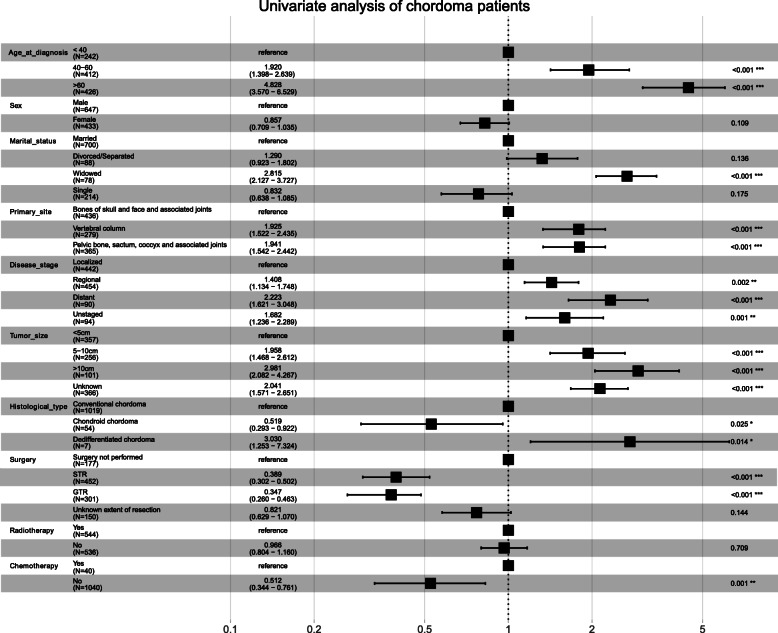
Forest plot of univariate Cox analyses of overall survival. The black squares on the transverse lines represent the hazard ratio (HR), and the transverse lines represent the 95% CI

**Fig. 5:**
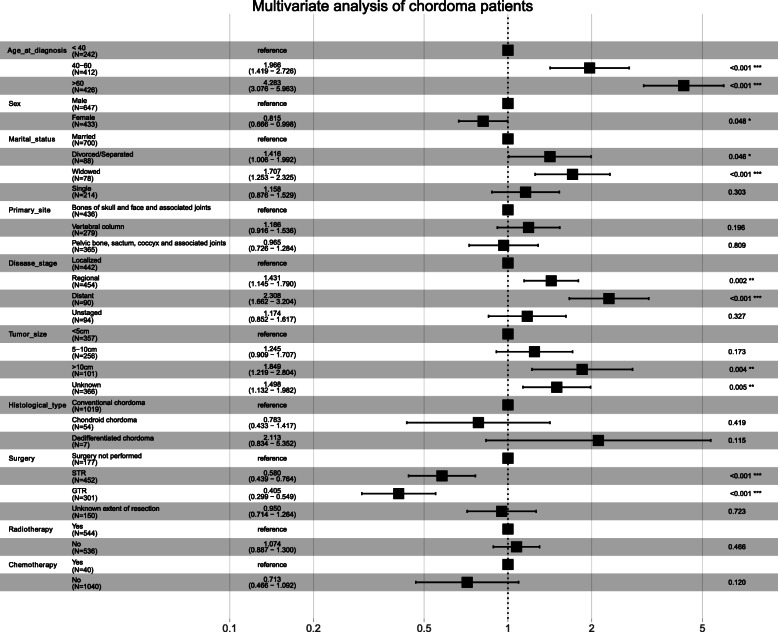
Forest plot of multivariate Cox analyses of overall survival. The black squares on the transverse lines represent the hazard ratio (HR), and the transverse lines represent the 95% CI

Univariate and multivariate Cox regression were used to analyze the prognostic factors associated with the OS of patients with chordoma (Table 4). Univariate Cox regression analysis showed that age at diagnosis, marital status, primary site, disease stage, tumor size, histological type, surgery, and chemotherapy were factors (all *p* < 0.05) related to OS in patients with chordoma (Fig. 4). Moreover, after all factors were included in the multivariate analysis, primary site, histological type, radiotherapy, and chemotherapy were not independent risk factors for patients with chordoma (Fig. 5). In addition, multivariate analysis showed that widowed patients had the worst OS (HR 1.71; 95% CI 1.25–2.33, *p* < 0.001) compared with married patients.

**Table 4:** Univariate and multivariate analysis of overall survival rates

Characteristic	Univariate analysis		Multivariate analysis	
	Hazard ratio (95% CI)	*p* value	Hazard ratio (95% CI)	*p* value
Age at diagnosis		< 0.001		< 0.001
< 40	Reference		Reference	
40–60	1.92 (1.40–2.64)	< 0.001	1.97 (1.42–2.73)	< 0.001
> 60	4.83 (3.57–6.53)	< 0.001	4.28 (3.08–5.96)	< 0.001
Sex				
Male	Reference		Reference	
Female	0.86 (0.71–1.04)	0.109	0.82 (0.67–1.00)	0.048
Marital status		< 0.001		0.006
Married	Reference		Reference	
Divorced/separated	1.29 (0.92–1.80)	0.136	1.42 (1.01–1.99)	0.046
Widowed	2.82 (2.13–3.73)	< 0.001	1.71 (1.25–2.33)	< 0.001
Single	0.83 (0.64–1.09)	0.175	1.16 (0.88–1.53)	0.303
Primary site		< 0.001		0.181
Bones of skull and face and associated joints	Reference		Reference	
Vertebral column	1.93 (1.52–2.44)	< 0.001	1.19 (0.92–1.54)	0.196
Pelvic bone, sacrum, coccyx, and associated joints	1.94 (1.54–2.44)	< 0.001	0.97 (0.73–1.28)	0.809
Disease stage		< 0.001		< 0.001
Localized	Reference		Reference	
Regional	1.41 (1.13–1.75)	0.002	1.43 (1.15–1.79)	0.002
Distant	2.22 (1.62–3.05)	< 0.001	2.31 (1.66–3.20)	< 0.001
Unstaged	1.68 (1.24–2.29)	0.001	1.17 (0.85–1.62)	0.327
Tumor size		< 0.001		< 0.001
< 5 cm	Reference		Reference	
5–10 cm	1.96 (1.47–2.61)	< 0.001	1.25 (0.91–1.71)	0.173
> 10 cm	2.98 (2.08–4.27)	< 0.001	1.85 (1.22–2.80)	0.004
Unknown	2.04 (1.57–2.65)	< 0.001	1.50 (1.13–1.98)	0.005
Histological type		0.004		0.124
Conventional chordoma	Reference		Reference	
Chondroid chordoma	0.52 (0.29–0.92)	0.025	0.78 (0.43–1.42)	0.419
Dedifferentiated chordoma	3.03 (1.25–7.32)	0.014	2.11 (0.83–5.35)	0.115
Surgery		< 0.001		< 0.001
Surgery not performed	Reference		Reference	
STR	0.39 (0.30–0.52)	< 0.001	0.58 (0.44–0.76)	< 0.001
GTR	0.35 (0.26–0.46)	< 0.001	0.41 (0.30–0.55)	< 0.001
Unknown extent of resection	0.82 (0.63–1.07)	0.144	0.95 (0.71–1.26)	0.723
Radiotherapy				
Yes	Reference		Reference	
No	0.97 (0.80–1.16)	0.709	1.07 (0.89–1.30)	0.466
Chemotherapy				
Yes	Reference		Reference	
No	0.51 (0.34–0.76)	0.001	0.71 (0.47–1.09)	0.120

*Abbreviations*: *STR* subtotal resection, *GTR* gross total/radical resection

In the stratification of primary site and disease stages (Table 5), univariate analysis showed that marital status was a risk factor for OS in the primary site of “bones of the skull and face and associated joints,” “vertebral column,” and “localized,” “regional,” and “distant” disease stages. In addition, multivariate analysis showed that marital status was an independent risk factor for the primary site of “bones of skull and face and associated joints” and “vertebral column.” Moreover, although marital status was not an independent risk factor for the prognosis of patients with chordoma at the disease stages of “localized,” “regional,” and “distant,” widowed patients were at higher risk of survival compared with married, divorced/separated, or single patients.

**Table 5:** Univariate and multivariate analysis of overall survival rates based on primary site and disease stage

Characteristic	Univariate analysis		Multivariate analysis	
	Hazard ratio (95% CI)	*p* value	Hazard ratio (95% CI)	*p* value
Primary site				
Bones of skull and face and associated joints		< 0.001		0.017
Married	Reference		Reference	
Divorced/separated	2.01 (1.14–3.56)	0.016	2.31 (1.26–4.22)	0.007
Widowed	4.33 (2.35–8.00)	< 0.001	2.34 (1.18–4.65)	0.015
Single	0.74 (0.45–1.21)	0.233	1.15 (0.67–1.95)	0.618
Vertebral column		< 0.001		0.011
Married	Reference		Reference	
Divorced/separated	0.81 (0.44–1.51)	0.514	0.94 (0.48–1.86)	0.863
Widowed	3.33 (2.11–5.26)	< 0.001	2.43 (1.44–4.12)	0.001
Single	0.66 (0.39–1.10)	0.111	0.82 (0.48–1.43)	0.488
Pelvic bone, sacrum, coccyx, and associated joints		0.064		0.277
Married	Reference		Reference	
Divorced/separated	1.26 (0.72–2.19)	0.423	1.24 (0.69–2.25)	0.477
Widowed	1.74 (1.12–2.71)	0.014	0.96 (0.57–1.60)	0.867
Single	1.36 (0.90–2.05)	0.140	1.54 (0.99–2.38)	0.056
Disease stage				
Localized		< 0.001		0.483
Married	Reference		Reference	
Divorced/separated	1.15 (0.62–2.17)	0.655	1.17 (0.60–2.29)	0.650
Widowed	3.54 (2.16–5.80)	< 0.001	1.54 (0.89–2.67)	0.126
Single	0.79 (0.48–1.29)	0.340	1.20 (0.70–2.06)	0.519
Regional		< 0.001		0.055
Married	Reference		Reference	
Divorced/separated	1.22 (0.77–1.93)	0.401	1.31 (0.81–2.11)	0.266
Widowed	2.80 (1.82–4.32)	< 0.001	1.95 (1.23–3.10)	0.005
Single	0.69 (0.45–1.04)	0.073	1.09 (0.71–1.67)	0.702
Distant		< 0.001		0.159
Married	Reference		Reference	
Divorced/separated	6.83 (2.25–20.73)	0.001	4.43 (1.19–16.53)	0.027
Widowed	2.78 (1.14–6.80)	0.025	3.14 (0.71–13.99)	0.133
Single	1.31 (0.69–2.48)	0.405	1.33 (0.58–3.04)	0.497

## Discussion

In this 42-year retrospective study, we conducted univariate and multivariate Cox regression analysis of a large number of adult patients with chordoma through the SEER database. We found that marital status was an independent risk factor for OS in adult patients with chordoma, and marital status had a protective effect on the survival outcome of adult patients with chordoma.

Marital status is widely considered to be an independent prognostic factor for many malignancies [17–20]. However, the effect of marital status on adult patients with chordoma has not been fully investigated. In this study, we first explored the effect of marital status on the OS of adult patients with chordoma, and we found that married patients had better OS than divorced/separated and widowed patients. In multivariate analysis, after adjusting for diagnosis age, sex, marital status, primary site, disease stage, histological type, tumor size, surgery, radiotherapy, and chemotherapy, marital status was still a risk factor for patients with chordoma. The widowed group patients had the highest risk ratio (HR 1.71; 95% CI 1.25–2.33, *p* < 0.001), and the benefits of married patients remained. Compared with the married, divorced/separated, or single groups, widowed patients had the worst 5-year OS (45.2%), 10-year OS (12.5%), and median survival time (56.0 months). Similar results were observed in the subgroup analysis of primary site and disease stages.

The effect of marital status on the survival of patients with chordoma has been studied before. Pan et al. [8] analyzed 808 patients with primary spinal chordoma from 1973 to 2014 and found that marital status was not the main factor affecting OS. Huang et al. [16] also showed that marital status was not a prognostic factor for patients with primary spinal chordoma. In our study, we included chordoma in the skull base, excluded all patients younger than 18 years old, and divided patients into four groups (married group, divorced/separated group, widowed group, and single group). It was found that marital status was an independent prognostic factor for adult patients with chordoma, which reduced the bias in case selection.

In our study, we found that the proportion of patients over 60 years old in the widowed group was as high as 94.9%, which was significantly higher than that in the married, divorced/separated and single groups. Elderly patients are more likely to die due to their poor physical quality and greater complications [21], which may be an important reason for the low survival rate of the widowed group. In addition, we also found that women accounted for the highest proportion (73.1%) of the widowed group. The activity of natural killer cells (NKs) plays an important role in the defense against tumors and virus infection. Studies have shown that bereaved women showed a decrease in NK activity and an increase in plasma cortisol levels compared with the control group, which may also lead to an increase in mortality in widowed patients [22].

In addition, the widowed group had the highest (37.2%) rate of non-surgery, and inadequate treatment may also lead to deterioration of the prognosis of the widowed group [23]. Moreover, widowed patients have an increased risk of stress and mental illness due to the lack of a partner [24]. In contrast, married patients have better family conditions and can receive more social support from their spouses and families [25]. Good marital status can help reduce anxiety, stress, and negative emotions and provide more material help. Studies have shown that negative emotions can lead to longer infection times and longer wound healing [26].

There are limitations to be recognized in this study. First, this study was a retrospective study with inevitable selection bias. Second, the SEER database only records marital status at the time of diagnosis, but it does not report whether the subsequent marital status has changed. This change will also affect the survival of patients and confuse the differences in survival outcomes based on marital status. In addition, the specific details regarding radiotherapy and chemotherapy were not included, such as the specific regimen of chemotherapy or the dose, fractionation, and beam energy of radiotherapy, which may also be prognostic factors for patients with chordoma.

## Conclusions

Our study found that marital status was an independent prognostic indicator for adult patients with chordoma and that marital status was conducive to patient survival. Widowed patients had worse OS than the other groups of patients, and similar results were observed in the subgroup analysis.

## Data Availability

The datasets generated for this study are available on request to the corresponding author.

## Abbreviations

SEER: Surveillance, Epidemiology and End Results
OS: Overall survival
CT: Chemotherapy
RT: Radiotherapy
HR: Hazard ratios
CI: Confidence interval
TNM: Tumor-node-metastasis
